# Development and use of a novel tool for assessing and improving researcher embeddedness in learning health systems and applied system improvements

**DOI:** 10.1017/cts.2023.667

**Published:** 2023-10-31

**Authors:** Nathan D. Shippee, Elisheva R. Danan, Mark Linzer, Helen M. Parsons, Timothy J. Beebe, Felicity T. Enders

**Affiliations:** 1 Division of Health Policy and Management, School of Public Health, University of Minnesota, Minneapolis, MN, USA; 2 VA HSR&D Center for Care Delivery and Outcomes Research, Minneapolis VA Healthcare System, Minneapolis, MN, USA; 3 Department of Medicine, University of Minnesota Medical School, Minneapolis, MN, USA; 4 Division of General Internal Medicine, Hennepin Healthcare, Minneapolis, MN, USA; 5 Department of Quantitative Health Sciences, Mayo Clinic, Rochester, MN, USA

**Keywords:** Learning health systems, embeddedness, research training, career development, hidden curriculum

## Abstract

This paper outlines the development, deployment and use, and testing of a tool for measuring and improving healthcare researcher embeddedness – i.e., being connected to and engaged with key leverage points and stakeholders in a health system. Despite the widely acknowledged importance of embeddedness for learning health systems and late-stage translational research, we were not aware of useful tools for addressing and improving embeddedness in scholar training programs. We developed the MN-LHS Embeddedness Tool covering connections to committees, working groups, leadership, and other points of contact across four domains: patients and caregivers; local practice (e.g., operations and workflows); local institutional research (e.g., research committees and agenda- or initiative-setting groups); and national (strategic connections within professional groups, conferences, etc.). We used qualitative patterns and narrative findings from 11 learning health system training program scholars to explore variation in scholar trajectories and the embeddedness tool’s usefulness in scholar professional development. Tool characteristics showed moderate evidence of construct validity; secondarily, we found significant differences in embeddedness, as a score, from baseline through program completion. The tool has demonstrated simple, practical utility in making embeddedness an explicit (rather than hidden) part of applied and learning health system researcher training, alongside emerging evidence for validity.

## Introduction

Learning health system (LHS) research has become a highly developed and supported paradigm in health services, clinical, and late-stage translational research. In particular, training programs and resources (e.g., competency lists) from the Agency for Healthcare Research and Quality (AHRQ) and Patient-Centered Outcomes Research Institute (PCORI) [1,2], as well as an orientation toward LHS work in Veteran’s Health Administration research training programs [3,4], have placed a knowledge premium on improving system structures and processes to obtain better patient, provider, and health system outcomes.

Embeddedness is widely acknowledged as a core piece of LHS research and training [5]. Embeddedness represents being located in, connected to, and responsively oriented toward the systems that researchers are trying to improve [6–8]. This helps researchers identify and address challenges of substantial interest to health systems (rather than setting a research agenda disengaged from practice concerns) [9,10]. Importantly, embeddedness also provides access to key leverage points (e.g., institutional resources and leadership support) to ensure that system improvements, and the research around them, can be successfully implemented [6,7].

Being “embedded,” therefore, means being engaged and connected with agents within and surrounding the systems in which researchers work. This creates rich sets of links to contacts and stakeholders in areas that affect the success or failure of implementation and maintenance of LHS improvements and research. Relevant agents include practice and research groups within one’s system, patients or caregiver groups, and national networks – important both for career development and for greater success in coordinating and diffusing innovations across systems [11]. As such, embeddedness is multi-factorial and likely to look different depending on the system one is trying to change and the definitions of “success” for each project and researcher. Because of this, training programs and resources aimed at improving embeddedness require flexibility to meet each LHS researcher’s needs.

Available training and resources for LHS researchers have generally focused on research and career competencies [12,13]: the “how-to” of intervention and research, including substantive knowledge (e.g., information systems), methods (mixed methods, implementation science), or theory (systems theory). These include competency lists and aforementioned training programs such as the AHRQ- and PCORI-funded LHS K12 training programs. Our own such program, the Minnesota Learning Health System Mentored Career Development Award (MN-LHS) program, previously published one such competency appraisal tool mapped to AHRQ competencies [14].

Despite the practical importance of embeddedness, we are not aware of any tools or resources oriented toward evaluating and improving researcher embeddedness. Others have also noted a need for explicit attention to embeddedness in training [15]. As leaders of an LHS training program, this meant that despite embeddedness as a “throughline” for our own program, it ran the risk of becoming hidden curriculum [16] – important, but not explicitly addressed. This under-prepares some while unfairly benefitting those “in the know.” Therefore, we developed, deployed, and tested a simple, adaptable embeddedness tool for training and career development.

In this paper, we describe the design and use of this embeddedness tool and present narrative and statistical findings on its use for scholar training.

## Materials and Methods

### Description of the MN-LHS Embeddedness Tool and Its Programmatic Use

We developed the tool iteratively at the initiation of the MN-LHS program in 2018. Alongside adapting and developing our competency appraisal tool, we realized the importance of intentionally increasing embeddedness both in our application for the training grant and tangibly for scholars trying to develop their careers and change systems. Based on expert consensus within our team regarding practice-based and healthcare delivery research and applied methods training, and focusing on simple applicability across scholar heterogeneity, we identified four domains:

- Patient (patient and family advisory groups) – because most system improvements will impact patient outcomes/experiences;
- Practice (practice committees and local/regional leadership) – for the practical changes to health systems and operations required for most LHS interventions/changes;
- Institution (research or agenda/initiative-setting committees) – for prioritization of projects and alignment with local initiatives, interests, and incentives; and
- National (national clinical and professional networks) – for career development, networking, and to support future diffusion of innovations.

Most changes to pilot versions were minor and involved creating anchor descriptions of scoring, wording for scores, and developing ways to use the tool (see below). One important consideration is that while the tool is intended to address a construct (embeddedness) related to career development, it is not a career development assessment per se. Alternatively, successful leadership showing high career development can be understood as just one type of high-level embeddedness, a related but distinct construct.

**Figure 1.**
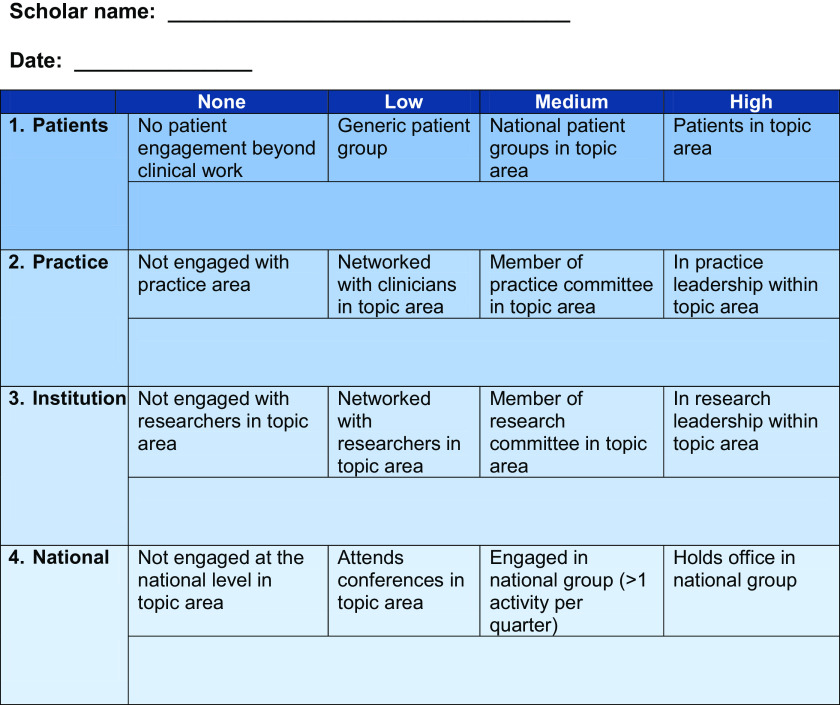
MN-LHS embeddedness tool. Embeddedness domains. Please circle or bold the appropriate level for each domain and describe specifics of your involvement, including names of groups and contacts, in the space below.

The embeddedness tool itself is a self-rating sheet (Fig. 1). For each of the domains (Patient, Practice, Institution, and National), scores were 0 (no engagement), 1 (low), 2 (moderate), and 3 (high), meaning a total score at a given time could range from 0 to 12. Anchors are included for low, moderate, and high engagement in each domain.

The MN-LHS Embeddedness Tool is one component of scholar assessment/planning and part of program evaluation. Its primary goal is to guide discussions in Individual Development Plan (IDP) meetings to plan how each scholar might become more embedded with Patients, Practice, Institution, and National domains. Its simple, face-valid design, with few components and easy scoring, was intended to facilitate part of the IDP discussion rather than measurement/tracking per se. The meeting/discussion process can be summarized as:

1. Program administration sends the embeddedness and competency [14] tools to the scholar.
2. Scholar fills out the tool electronically, including scoring each domain item and writing in current/planned engagement, committee, or other activities to meet each of the domain goals.
  - The scholar also fills out our LHS competency tool to identify and compare current progress with AHRQ LHS research competencies and PCORI methods standards (cite).
3. Scholar sends the tool back to the program administrator prior to each IDP meeting
4. IDP meeting with curriculum directors, scholar, and mentor(s) as needed
  - Discussion with scholar and mentor on project/learning progress
  - Guided discussion with competency and IDP tools to identify and brainstorm strategic and practical steps for career and project advancement
  - Enlisting mentors in planning and problem-solving, including explicitly requesting sponsorship to aid the scholar’s embeddedness

Part of the IDP meeting discussion does examine growth in research competencies and progress on projects/learning objectives, but discussions are primarily centered on increasing embeddedness aligned with the scholar’s research and career aspirations. However, as we have continued to administer the program in leadership and mentoring roles, we have developed several tactical facets of use for the embeddedness tool, within the following non-exhaustive list:

1. Activating sponsorship
  - Formal training for program mentors
  - Guiding scholars to potentially supportive network connections
2. Alignment and justification of embeddedness roles with career goals
  - Embeddedness roles (e.g., committees, national conference work) that have a service component are aligned with the overall strategy
  - Discussions help ensure that national, local/regional, and other embeddedness roles/activities will move the scholar toward the level they want
3. Ensuring women and minoritized scholars get directed attention centered on their needs and/or regarding barriers unique to their experiences
  - Gendered socialization, lack of group-concordant mentors and spaces, potential imposter syndrome, and other challenges act as systemic and sometimes personal barriers that can be addressed with a combination of mentorship, sponsorship, networking, and guided training
  - Women and minoritized scholars often have needs beyond those met by the research mentor. Such needs frequently arise in discussions of embeddedness, as at minimum, embeddedness entails inclusion. When warranted by discussion, issues with inclusion were addressed through either 1) sponsorship, wherein program leadership requested that the mentor initiate inclusion of the scholar in a specified activity, or 2) direct intervention, when the scholar’s behaviors suggested a lack of confidence, comfort, or belonging. The latter often arose among individuals previously socialized to act in a minoritized manner; such behaviors can be changed through coaching [17,18] and explicitly uncovering the hidden curriculum [16].

Examples of specific steps taken included the following: scholars requesting temporary positions on key committees or shadowing (Practice domain), and narrowing and focusing on specific conferences or organizations to develop a presence and core network (National). We have found it useful to direct scholars to explain that the program requires certain experiences when making requests for committees, shadowing, etc., which can make the requests less intimidating to the scholar. We also use these discussions to nudge mentors to act as sponsors to the scholar in ways aligned with the scholar’s needs.

As described above, we have found the tool useful during individual scholar development planning. We also wanted to examine heterogeneity in scholar trajectories and to understand if the tool was supportable as valid, sufficiently to be applied elsewhere.

### Sample

Our sample consisted of 11 scholars with both baseline and “graduation”/completion scores on the embeddedness tool across 5 cohorts of the MN-LHS training program. Scholars were administratively located across 4 institutions in Minnesota. Observation years covered Fall 2018-Spring 2023.

### Embeddedness Measurement

The measure consisted of the embeddedness tool as a summary score across the four domain items, with each domain item scored from 0-3 and the summary ranging from 0-12, with higher scores indicating higher embeddedness.

We descriptively examined each scholar’s trajectory in terms of changes in roles, leadership opportunities, committee and collaborative positions, engagement with patient groups, national conferences, and other representations of the four domains.

### Analysis

To examine scholars’ trajectories, we used narrative qualitative descriptions of scholar experiences. Scholars in different systems and specialties had varied needs and diverse trajectories, insufficiently captured by scores alone.

We also used construct validity criteria [19] to establish validity of the tool. While not the central focus of this paper, we believed it would allow critical examination by administrators of other similar training programs in LHS and late-stage translation.

Lastly, we examined quantitative differences in baseline versus program completion embeddedness. Although the tool was not designed/used as an outcome “score,” we tested pre-post-differences for transparency and to support basic construct validity. With a total possible range from 0 to 12 at each time-point, we examined differences in embeddedness “score” at baseline versus completion of the program (one scholar scored themselves as midway between two levels at baseline; we used the lower level for their score). To assess differences in summary scores, we used the Wilcoxon signed rank test to compare the within-scholar difference in summary embeddedness score to the null hypothesis of no change in score.

## Results

### Narrative Qualitative Findings

**Figure 2.**
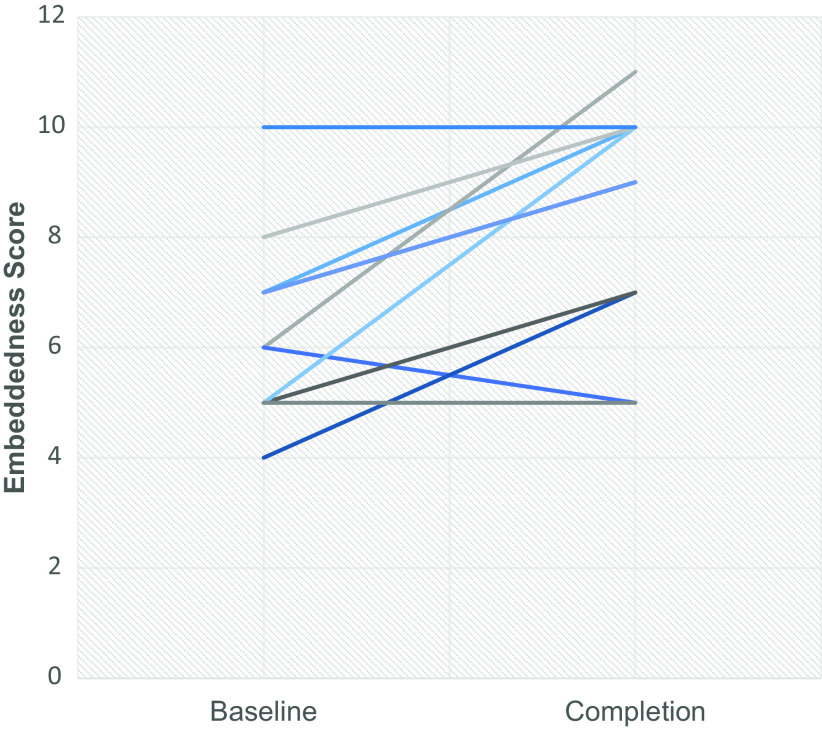
Variation in change in embeddedness score, baseline to completion. *N* = 11.

Patterns in scholars’ embeddedness were heterogeneous, with baseline/completion trends varying from −1 to + 5 (see Fig. 2). However, scores did not adequately capture scholars’ experiences.

Narratively, the individual with a change of -1 was a PhD who began the program with a Patient domain score of 1 because they were engaged with a generic patient group, along with high engagement in Practice and National in their field (primarily operations research). IDP discussions established that this engagement was not helpful to them and that a better use of time in this scholar’s case was strategically focusing on activities toward national reputation in health services research specifically for a better position toward promotion. Consequently, this change in score was actually viewed as a positive outcome and did not reduce other forms of engagement.

While on average, scholars were 2 points higher on embeddedness at completion versus baseline, individual trajectories represented various baseline and completion circumstances. For example, one PhD scholar (baseline 5, completion 7) began the program with Practice and Institution scores of 1 each because they were networked with both clinicians and researchers in their topic of pharmacy health outcomes. During the program, they started attending the monthly clinical pharmacist meeting and became part of a diabetes education integration workgroup, shifting the Practice score from 1 to 2. They also joined the “Tier 3 Research and Education” committee, which is in charge of achieving education and research goals for the primary care service line at their institution’s health system, thereby shifting her Institution score from 1 to 2.

Meanwhile, another PhD scholar with a “change” of + 2 (baseline 7, completion 9) also began the program with an Institution score of 1, because they were networked with other clinicians and researchers through his mentor’s AHRQ R01. During the program, they were appointed as Vice President for Equity in Research at their institution. Through this position, they are now responsible for developing and supporting efforts to build Hennepin Healthcare Research Institute’s (HHRI’s) capacity to conduct community-engaged and health disparities research and promoting equity in the practice of research focusing on participant-oriented and workforce concerns. The position also conveys membership on the institution’s Operations Team and Board of Directors. As a result, the Institution score shifted from 1 to 3.

In contrast, two individuals had larger baseline/completion differences of + 5 points, again unique to each scholar. One, an MD with a baseline 5 and completion embeddedness 10, achieved leadership in the Practice, Institutional, and National levels during the program. Highlights of his current engagement include becoming a leader of the implementation of a pharmacogenomics program at his clinical institution, establishing an institutional collaboration with an outside testing entity to test pharmacogenomics microarray, and joining the organizing committee for his institution’s pharmacogenomics Conference (leading Institutional work aligned with his topic), and elected as a member of the Association for Molecular Pathology’s Clinical Practice Committee and Hematopathology working group and invited to join the national Gene Product Nomenclature Consortium (leading national work aligned with his topic).

### Construct Validity

Although face validity seemed high and adapting the tool across scholars’ needs (as opposed to measurement) was paramount, we examined construct validity using criteria from Cook and Beckman [19] to establish whether other training programs might consider the tool sufficiently broadly valid (and not over-fitted to one local context or program). We address each construct validity criterion below.

1. Expert creation of items. The team developing and using the tool with scholars has decades of experience in teaching research methods across quantitative, qualitative, mixed, implementation, and other methods areas; conducting healthcare delivery and late-stage translational work under national and local system funding sources; and participating in mixed-training team science with practitioners and academics. They also collectively have had several leadership positions across multiple training programs including lead directorships, PI/MPI arrangements, curriculum directorships, etc., and have served on multiple national grant study sections. Lastly, they have mentored dozens of scholars across pre- and post-doctoral programs.

2. Easy to answer questions. The items used in the tool (Fig. 1) are brief and are self-rated with anchor examples provided to help the tool be intuitive. In addition, each domain rating (four in total) addresses the same underlying question: “At what level am I?”

3) Reliability/internal consistency. This criterion is the least easily supported given the brevity of the instrument, its design, and its intended use. Specifically, each domain is varyingly important across scholars (i.e., they have different needs/goals), and the instrument is self-rating on only four domains. Likewise, the instrument’s test-retest reliability is of questionable usefulness given the intention for growth over time, and the intraclass correlation coefficient estimation that might address within/between differences over time would likely require a larger sample to be informative.

4) Convergent validity (alignment with a theoretically plausible outcome). As shown above, scholar narratives provide qualitative evidence indicating larger growth on the embeddedness tool (e.g., an increase of + 5) is qualitatively aligned with more extensive career advancement, local and national network connections, etc.

5) Positive consequences: Are those using it better off for the instrument? Given our small sample and the potential for various biases in rating the scholars of our program, this criterion requires circumspection. We can say that completed scholars have gone on to administrative leadership, training program leadership, gained tenure, built team science networks that have led to multiple R-level grants, and other successes requiring extensive or healthy networks. In addition, the tool’s benefit in rendering embeddedness as an explicit part of training and providing a language and placeholder for discussion in IDP meetings has also been helpful in facilitating intentional focus on embeddedness.

In sum, we believe that, based on limited evidence, the tool at present is arguably supported by evidence for 4 of 5 construct validity criteria.

### Statistical Findings

While acknowledging concerns of both authors and reviewers regarding the presentation of statistical results as falsely indicative of “effectiveness” (establishment of which was not an aim in the tool’s development), we did statistically compare baseline and completion embeddedness “scores” (see Appendix A). Of 11 scholars with embeddedness data at baseline and program completion, mean embeddedness at baseline was 6.63 (standard deviation = 1.69) and at completion was 8.64 (standard deviation = 1.86), with the aggregate difference statistically significant. Of course, increases in these summary scores mask many qualitative changes noted above and could represent, for instance, small differences in two domains or a large difference in a single domain. Moreover, examining such scores in the aggregate does not favor the central goal of working with scholars on their individual development plans to strategically position/engage.

## Discussion

The MN-LHS Embeddedness Tool’s usefulness in improving communication in two directions (helping the program team communicate priorities and needs to scholars while also helping scholars communicate their contexts and incentives to the program team) has been invaluable in making embeddedness an *explicit* part of our training program, rather than hidden curriculum. As embeddedness is a difficult topic to teach given the context-dependent and scholar-background heterogeneity involved, this kind of tool helps provide language and discussion points for optimal communication, shared mental models, and planning. And, as shown, we have found useful ways for this tool to inform scholar development and strategic investment of time into activities and connections.

The development and evaluation of this tool has limitations. The limited number of scholars from one program in a geographically limited area may limit generalizability of narrative findings or experiences to other scholars. Similarly, more extensive analyses become difficult with such a small sample of completed scholars. As such, our statistical analysis does not adjust for key covariates – nor for key mediators or mechanisms that may explain the change in embeddedness (e.g., mentor actions, system leadership of organizations). However, our primary interest – evaluating whether the embeddedness tool is practically useful – appears supported to move forward with its use while conducting additional evaluative or developmental work). Similarly, our construct validation does rely on narrative and qualitative or mixed qualitative-quantitative evidence primarily. This is partially because career development, embeddedness, and other constructs that might help further validate through additional formal convergent or discriminant validity do not generally have readily available measures. Yet, our narrative results indicate real experiences of growth within the program toward closer engagement (with noted variations), and other construct validity criteria are mostly well-supported.

Despite limitations, key takeaways from both this analysis and our use of the embeddedness tool have been:

- Many roles and opportunities for embeddedness fall under the general categories of “stakeholder engagement” or “service,” and so come with a cost in time, effort, and responsibilities.
  - Past scholars have explicitly found this tool to be useful in answering questions such as “Should I serve on that committee, help with that conference, work with that patient group?” and similar questions toward building a better, healthier strategy.
- To the degree that embeddedness remains a hidden curriculum, it has serious implications for equity and access to quality training programs.
  - Health and healthcare equity and justice have become an LHS competency area [1]; if training programs are to be internally consistent with this, explicit attention to authentic embeddedness efforts must be integral.
- There are wide variations in the ways researchers can become more embedded [7,8].
  - These depend on professional training and licensure, direct/indirect contact with the clinical space as a clinician versus academic scholar, systems in which scholars are embedded, and other factors. A standardized, but flexible, approach is needed in training tools.
- Embeddedness is part of, or similar to, two separate concerns: engagement and professional development, but is conceptually and practically distinct.
  - Engagement must happen and be sustained to establish embeddedness.
  - Embeddedness is partially reliant on, and also typically necessary for, successful professional development; as noted above, good leadership can be seen as requiring or representing high-level embeddedness.

## Conclusion

Difficult concepts in LHS and LHS training can be operationalized into actionable tools to ensure project success, improve systems, and advance careers. The MN-LHS Embeddedness Tool is simple, broadly applicable, and useful in discussing and assessing current state embeddedness and in planning career development goals to position scholars for success, has moderate evidence for construct validity, and modest statistical evidence of scholar improvements after completion of a LHS training program. This helps to meet the identified need for explicit attention toward embeddedness in training programs (rather than remaining a hidden curriculum) [15], with the end goal being the continued and expanded improvement of systems and outcomes.

## Supporting information

Shippee et al. supplementary material Shippee et al. supplementary material

## Supplementary material

The supplementary material for this article can be found at https://doi.org/10.1017/cts.2023.667.
