# Supplementary material for: Development and use of a novel tool for assessing and improving researcher embeddedness in learning health systems and applied system improvements
Source: J Clin Transl Sci. 2023 Oct 31;7(1):e248. doi: 10.1017/cts.2023.667 (PMC10789988; doi:10.1017/cts.2023.667)
Supplement: Shippee et al. supplementary material [file S2059866123006672sup001.docx]

Appendix A.

Figure: Differences in embeddedness tool as an overall “score,” from baseline to program completion (generally two years).

difference p=.0018

Notes:

1. Scores represented baseline and completion scores on the MN-LHS Embeddedness Tool.

2. Baseline and completion scores ranged from 0-12; total possible range in change from baseline was -12 to +12.
